# Supplementary material for: Large-scale estimation of bacterial and archaeal DNA prevalence in metagenomes reveals biome-specific patterns
Source: mSystems. 2026 Mar 19;11(4):e01062-25. doi: 10.1128/msystems.01062-25 (PMC13098197; doi:10.1128/msystems.01062-25)
Supplement: Supplemental Information — Notes S1-S5, Tables S1 and S2, and Figures S1-S6. [file msystems.01062-25-s0001.docx]

# Supplementary Information

## Supplementary Notes

### Supplementary Note 1

We tested the performance of SPF under extremely challenging scenarios, when simple communities containing novel lineages were present (**Supplementary Figure 1**). In these scenarios, simulated communities contained two microbial species at equal abundance (10X coverage each), one present in both R207 and R214 releases of GTDB, and another only in R214. In each simulated dataset, the new genome had a different level of taxonomic novelty (from species to phylum) relative to R207. These metagenomes were analysed using SPF backed by the GTDB R207 database. SPF estimates were within 20% of the true value of 100% for most communities containing novel species (80%), genera (95%) and families (85%), but was less accurate for novel orders (55%), classes (60%) and phyla (70%). We consider this final set of benchmarks especially challenging since the communities contained very high quantities of very novel lineages. Communities dominated by species novel at the order level are increasingly rare as genome databases move towards completion, and in these situations combining SPF with a genome-centric approach might be appropriate (**Supplementary Note 2**).

### Supplementary Note 2

Seven hyena faecal samples yielded nonsensical SPF estimates over 100%. We found this was due to the actual genome sizes of one or a handful of species with high relative abundance (*i.e.* a highly uneven community) being much smaller than the mean genome size of their taxonomy in the GTDB. For example, sample G3_P301 had a highly abundant member classified by SingleM as g__Bacteroides, of which the mean genome size from the GTDB was 5.1 Mbp. The actual MAG size for this Bacteroides genome was 2.2 Mbp, resulting in >2.3 fold overestimation of its read contribution. We consider these scenarios to be rare, but have implemented a warning for users when a sample's SPF estimate could under- or overestimate the read fraction by >10%. Users are warned if the 3 highest abundance lineages not classified to the species level would change the estimated read fraction of the sample by >2% if their genome size is halved or doubled. In these situations, users should explore the samples that yielded warnings, and can, for example, update the SingleM estimates using genome sizes from MAGs that they generate to obtain more accurate estimates. In fact, when updating the estimates with MAG sizes from the hyena dataset, overestimations were drastically reduced (**Supplementary Figure 2**).

The most likely metagenomes to give rise to these situations are those dominated by a small number of highly abundant species. While it is challenging to estimate the microbial read fraction in these samples, MAG recovery from these samples is typically more successful since there is sufficient coverage and a comparatively less diverse community. The SPF algorithm warned about potential inaccuracies arising from species which were both highly dominant and novel in only 0.28% of public metagenomes, showing such cases are relatively uncommon in practice.

### Supplementary Note 3

A small minority of soil samples had unusually high STAT values and low SPF values (200 samples, 4.8%, **Figure 3F**). Upon further investigation, it was found that 85% of these samples were derived from two studies that used the same tagmentation library preparation protocol. We downloaded the sequencing data for these bioprojects to estimate the insert sizes, and found a median insert size of 92 (± 23.3), suggesting that the library preparations in the original studies were not optimised (important for tagmentation-based protocols) (**Supplementary Figure 5**). Since SingleM relies on 20 amino acid (60 nucleotide) sequences, and these sequences must be contained within stretches of at least 72 bases uninterrupted by stop codons, libraries with short insert sizes would deflate SingleM prokaryotic fraction estimates. For more details on the analysis and reproducible code, see <https://github.com/EisenRa/SingleM_microbial_fraction_paper/blob/main/code/SI_note2.md>

A small minority of soil metagenomes had very small SPF values. To investigate these, a randomly selected subset of 5 soil samples with SPF values <10% were manually inspected. Of these, 2 were viral enrichments, 2 were fosmid libraries and 1 was incorrectly classified as a soil metagenome. Therefore, in all 5 cases, SPF did not estimate erroneously low prokaryotic fractions. Instead, the samples analysed were not standard soil metagenomes.

### Supplementary Note 4

To test the performance of SPF’s ability to estimate prokaryotic Average Genome Size (AGS) from metagenomes with eukaryotic DNA, we used the simulated metagenomes from **Figure 1**. MicrobeCensus was run alongside to compare the performance of a current state-of-the-art tool. Both tools performed well on the Zymo mock community (mostly composed of bacteria) (**Supplementary Table 1**). However, when simulated eukaryotic reads were added to the Zymo mock community metagenome, MicrobeCensus severely overestimated prokaryotic AGS (**Supplementary Table 1**), while SPF values remained virtually unchanged. These findings are most likely due to MicrobeCensus’ assumption that all reads in metagenomes are microbial, which, as we show in the main text on thousands of publicly available metagenomes, is often untrue.

Using SPF, we surveyed the AGS of different environments by interrogating samples associated with different environments. Substantial differences were seen in between environments (**Supplementary Table 2**). The AGS of human oral samples was surprisingly low (2.43 Mbp), especially compared to human gut samples (3.48 Mbp).

### Supplementary Note 5

To investigate the potential for inaccuracy of SPF’s prokaryotic fraction and AGS in soil metagenomes, which provide a particular challenge to estimating the microbial read fraction because such few species present in them have genomic representation [(Woodcroft et al. 2025)](https://paperpile.com/c/64oTyf/zSVS). On its own, this does not necessarily lead to inaccuracy because the high alpha diversity of soils means that an overestimated genome size estimate for one lineage will likely be balanced by the underestimation of another. However, the average genome sizes of soil microorganisms are substantially higher than those of other ecosystems such as marine or host-associated systems (**Supplementary Table 2**). We investigated whether the paucity of genomes from soil bacteria and archaea in the reference databases combined with the high genome sizes observed in soil bacteria and archaea may lead to a systematic underestimation of prokaryotic fraction by the SPF algorithm. To interrogate this possibility, we analysed several soil-related datasets, looking for evidence of systematic underestimation of AGS by the SPF algorithm.

We first analysed those genomes from isolate cultures originally derived from soil environments. Specifically, GTDB species representatives were linked to BacDive [(Schober et al. 2025)](https://paperpile.com/c/64oTyf/sm94) isolated organisms, taking one example per species cluster. Overall, the mean of these genome sizes was 6.0 ± 2.4 Mbp. However, the distribution of genome sizes was bimodal, with peaks at ~4.1 Mbp and ~8.2 Mbp (**Supplementary Figure 6**). Closer inspection revealed that the larger of these peaks was dominated by isolates from the phylum Actinobacteria. The average Actinobacterial isolate genome size was 7.2 ± 2.5 Mbp, while the average of genomes derived from other phyla was much smaller, at 5.0 ± 1.9 Mbp. While common components of soils, Actinobacteria are overrepresented in the set of isolated soil organisms (45% of those species analysed here, 22.9% in soil communities recorded in Sandpiper [(Woodcroft et al. 2025)](https://paperpile.com/c/64oTyf/zSVS)). Therefore we conclude that the overall genome size mean (6.0 Mbp) is probably an overestimate driven by large actinobacterial isolate genomes. However, even if Actinobacterial genomes are excluded, the larger average genome size (5.0 Mbp) compared to the mean AGS in soil metagenomes estimated by SPF (4.41 Mbp) suggests that SPF may underestimate the AGS of soil communities.

We next analysed the set of MAGs in the SMAG dataset, a large collection of 40,039 genomes derived from soils [(Ma et al. 2023)](https://paperpile.com/c/64oTyf/BIfN). The average genome size of these MAGs was 4.0 ± 1.8 Mbp after adjusting their sizes based on CheckM2 estimates of completeness and contamination and including only those which were >50% complete and <10% contaminated (19,425 MAGs). This average is smaller than SPF’s estimate (4.4 Mbp) which suggests that SPF does not always underestimate AGS in soil metagenomes.

Finally, we analysed a metagenome dataset derived from soil microbial communities from Luquillo Experimental Forest, Puerto Rico [(Riley et al. 2023)](https://paperpile.com/c/64oTyf/RfTu). These metagenomes are very large, totalling 3.4 Tbp of sequence data, and MAGs have been recovered based upon the scalable MetaHipMer2 assembler. Across the samples, SPF estimated the prokaryotic fraction as 62.5 ± 2.6% and AGS 5.26 ± 0.04 Mbp. Adding a dereplicated set of genomes reported by Riley et. al. estimated by CheckM2 to be >50% complete and <5% contamination (223 genomes) to the SingleM R220 reference database with “singlem supplement”, the prokaryotic fraction estimate was increased to 63.9 ± 2.5% and the AGS increased to 5.39 ± 0.06 Mbp. This suggests that the original SPF AGS is likely to be an underestimation of the true AGS, though the quantity of the SingleM profile which could be assigned to the species level only increased from 8.3 to 11.8% on average with the addition of the new genomes, showing that the vast majority of species in these samples remain without species-level reference genomes.

##

## Supplementary Tables

**Supplementary Table 1.** Comparison of Average Genome Size (AGS) predicted by Microbe Census and SingleM (i.e. SPF) of samples containing Zymo mock data and various quantities of simulated Eukaryotic DNA sequences. True AGS of Zymo mock is 3,356,613 and % values are of true AGS value. When Eukaryotic sequences are added, The MicrobeCensus AGS estimates incorrectly go up, while the SingleM AGS correctly remains stable.

|  | **MicrobeCensus AGS** | **SingleM AGS** |
| --- | --- | --- |
| **Zymo mock** | 3,448,030 (102.7%) | 3,338,881 (99.5%) |
| **+ *Plasmodium* reads** | 5,175,322 (154.2%) | 3,337,578 (99.4%) |
| **+ *Arabidopsis* reads** | 40,565,012 (1,208.5%) | 3,331,983 (99.3%) |
| **+ *Homo* reads** | 388,505,521 (11,574.3%) | 3,338,970 (99.5%) |

**Supplementary Table 2.** SingleM Average Genome Sizes (AGS) of metagenomes found in various habitats, in decreasing order of mean. Soil metagenomes with an SPF < 5% were excluded from these statistics.

| **Environment** | **AGS mean** | **AGS s.d.** | **AGS median** | **Sample #** |
| --- | --- | --- | --- | --- |
| plant | 4.95 | 1.36 | 4.78 | 520 |
| soil | 4.41 | 0.56 | 4.40 | 4,590 |
| human gut | 3.48 | 0.68 | 3.37 | 34,321 |
| food | 3.26 | 0.80 | 3.29 | 1,115 |
| marine | 2.60 | 0.93 | 2.28 | 6,959 |
| human oral | 2.43 | 0.42 | 2.37 | 1,031 |

##

## Supplementary Figures


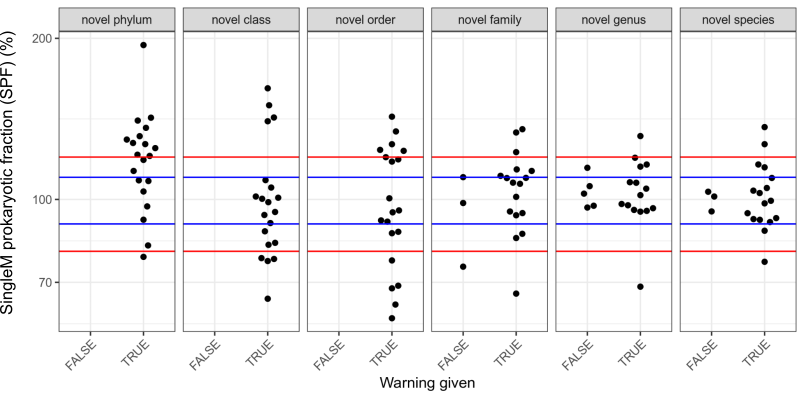


**Supplementary Figure 1**. Two-component community accuracy and whether a warning of an unreliable estimate is provided by SPF. Each dot represents a community made up of two components at equal coverage, one species present in GTDB R207, and the other new in GTDB R214. The community was analysed using an R207-based database, such that the species new in R214 was novel at the taxonomic level indicated in the title of each panel. The true value is 100% in all cases since the communities are exclusively comprised of prokaryotic lineages. This benchmark is extremely challenging, since the genome sizes of unknown lineages is difficult to estimate. We see that a warning about an unreliable estimate is given in most cases, and most of the SPF values are within the 80-120% range indicated by the horizontal red lines. Red lines represent the 90-110% range. Values above 100% are shown here for the purposes of benchmarking, but in practice SPF values are capped at 100% since higher values are impossible. These results are discussed in **Supplementary Note 1**.


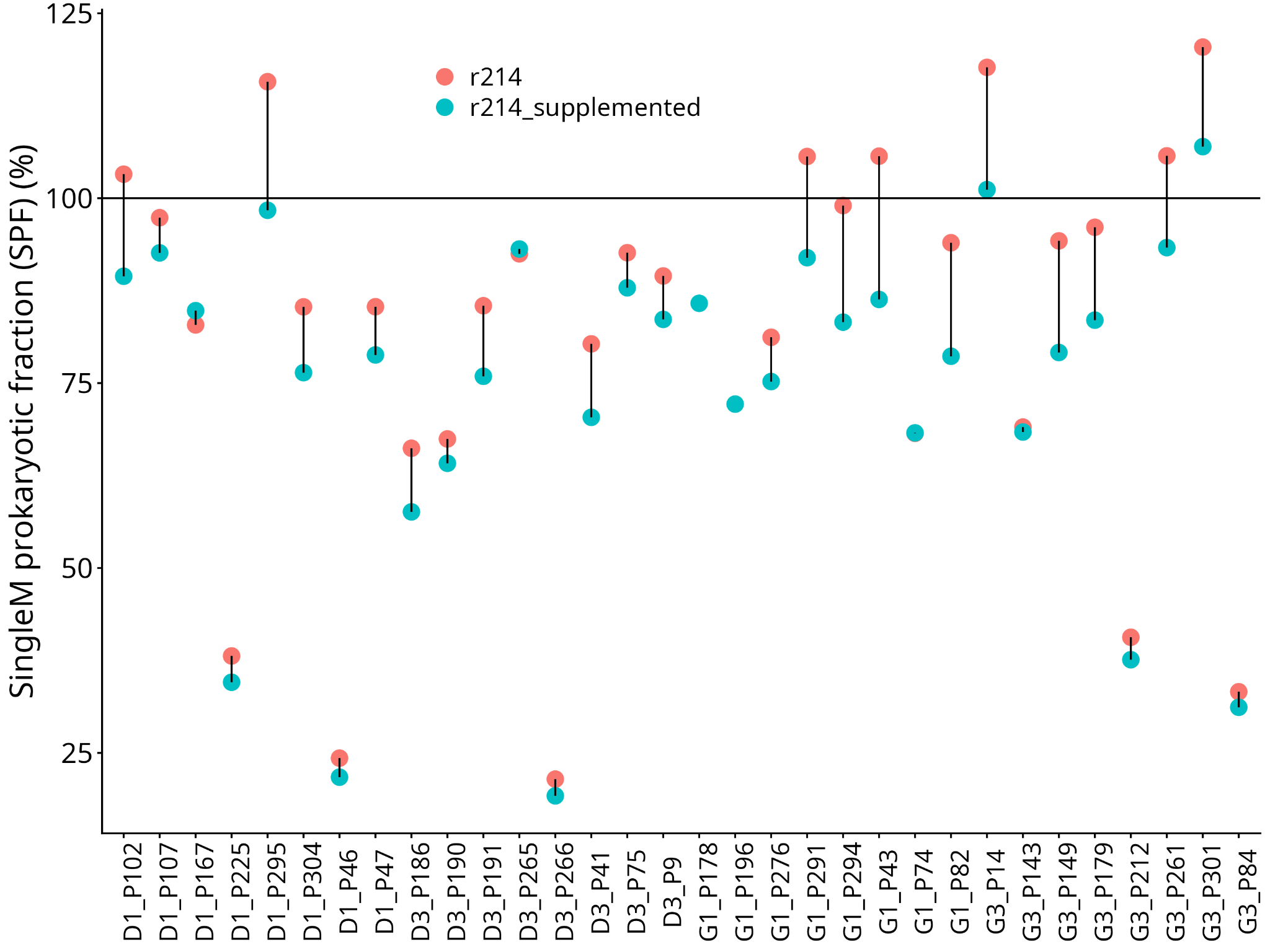


**Supplementary Figure 2**. SingleM prokaryotic fractions of hyena faecal samples with default GTDB R214 genome sizes (red), and genome sizes supplemented with MAGs (blue).


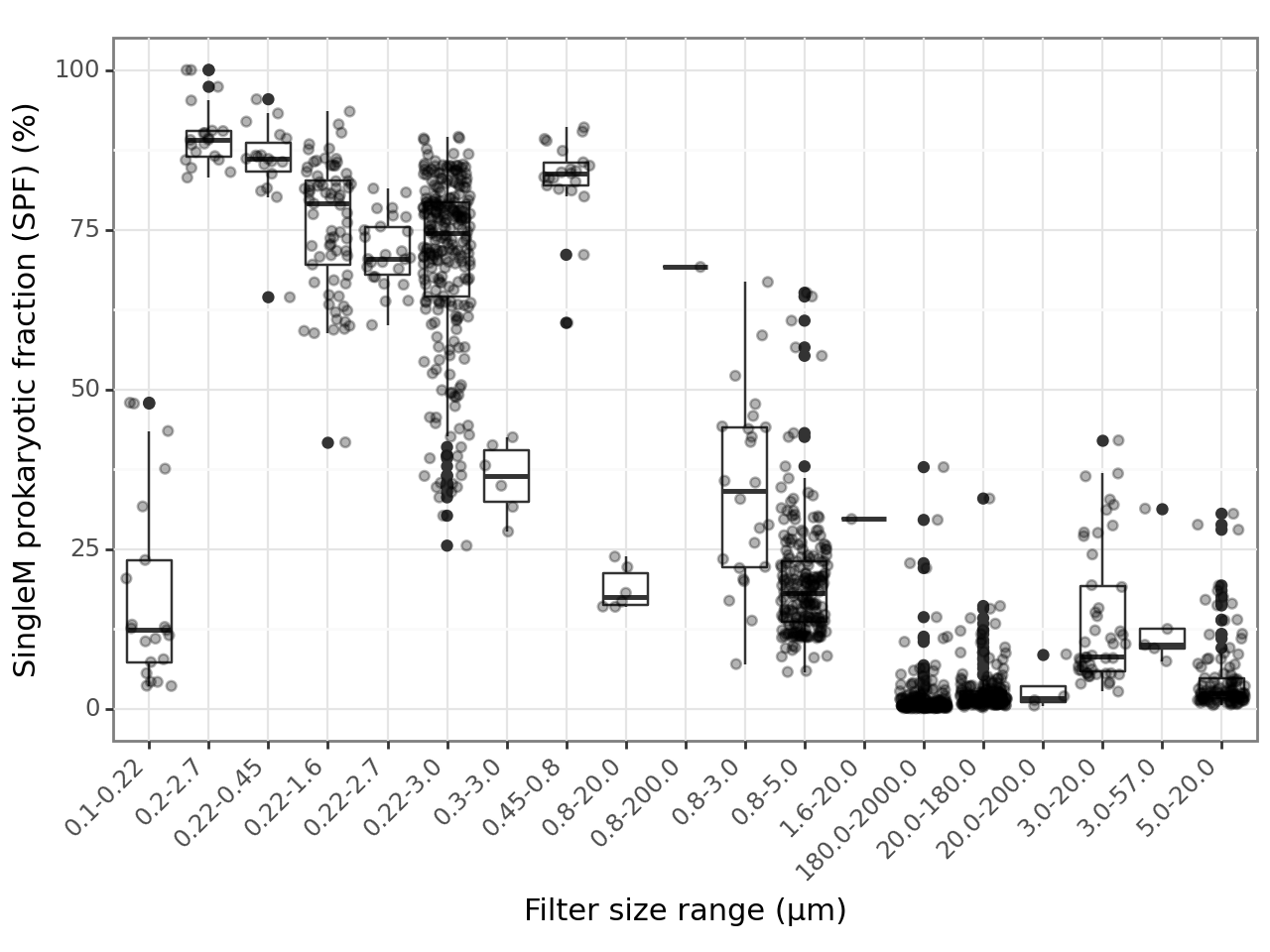


**Supplementary Figure 3. Size fractionation influences microbial read fraction in marine metagenomes.** Sequential size filtration is commonly used to separate cells of different sizes in the analysis of marine metagenomes. Where available as part of biosample metadata [(Sunagawa et al. 2015; Pascoal et al. 2023; Hevroni et al. 2020; Hugerth et al. 2015)](https://paperpile.com/c/64oTyf/UtNb+ESMf+jUmH+9O7l), filter sizes (upper and lower sizes) were parsed and used here to stratify SingleM prokaryotic fraction (SPF) estimates in marine metagenomes. SPF estimates were substantially affected by filter size, where the application of intermediate size bands between 0.22 and 0.8 μm resulted in higher SPF values, compared to smaller and larger filter sizes. This observation is consistent with established practice, since these smaller and larger sizes are thought to enrich for viral and eukaryotic cells, respectively, while intermediate sizes are thought to enrich for Bacteria and Archaea.


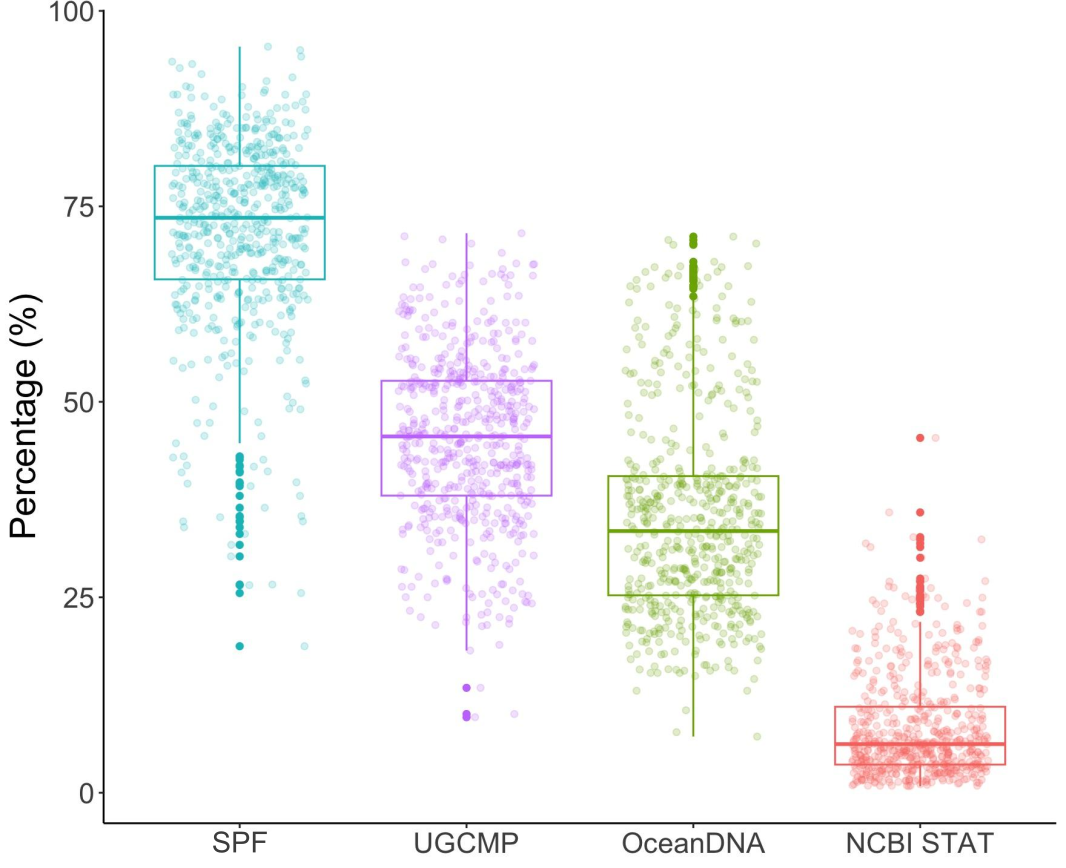


**Supplementary Figure 4**. Use of SingleM Prokaryotic Fraction (SPF) to estimate the representativeness of marine genome catalogues. SPF values estimate the percentage of reads in the metagenome which are prokaryotic, and so represent a maximal read mapping rate achievable to the following reference databases / methods, which only account for prokaryotic genomes (mapping artifacts aside). UGCMP = mapping rate of samples to the Unified Genome Catalog of Marine Prokaryotes. OceanDNA = mapping rate of samples to the OceanDNA MAG catalogue. STAT = STAT estimate of metagenome prokaryotic fraction. Mapping rates were obtained from Nishimura et al. 2022 supplementary table 1.


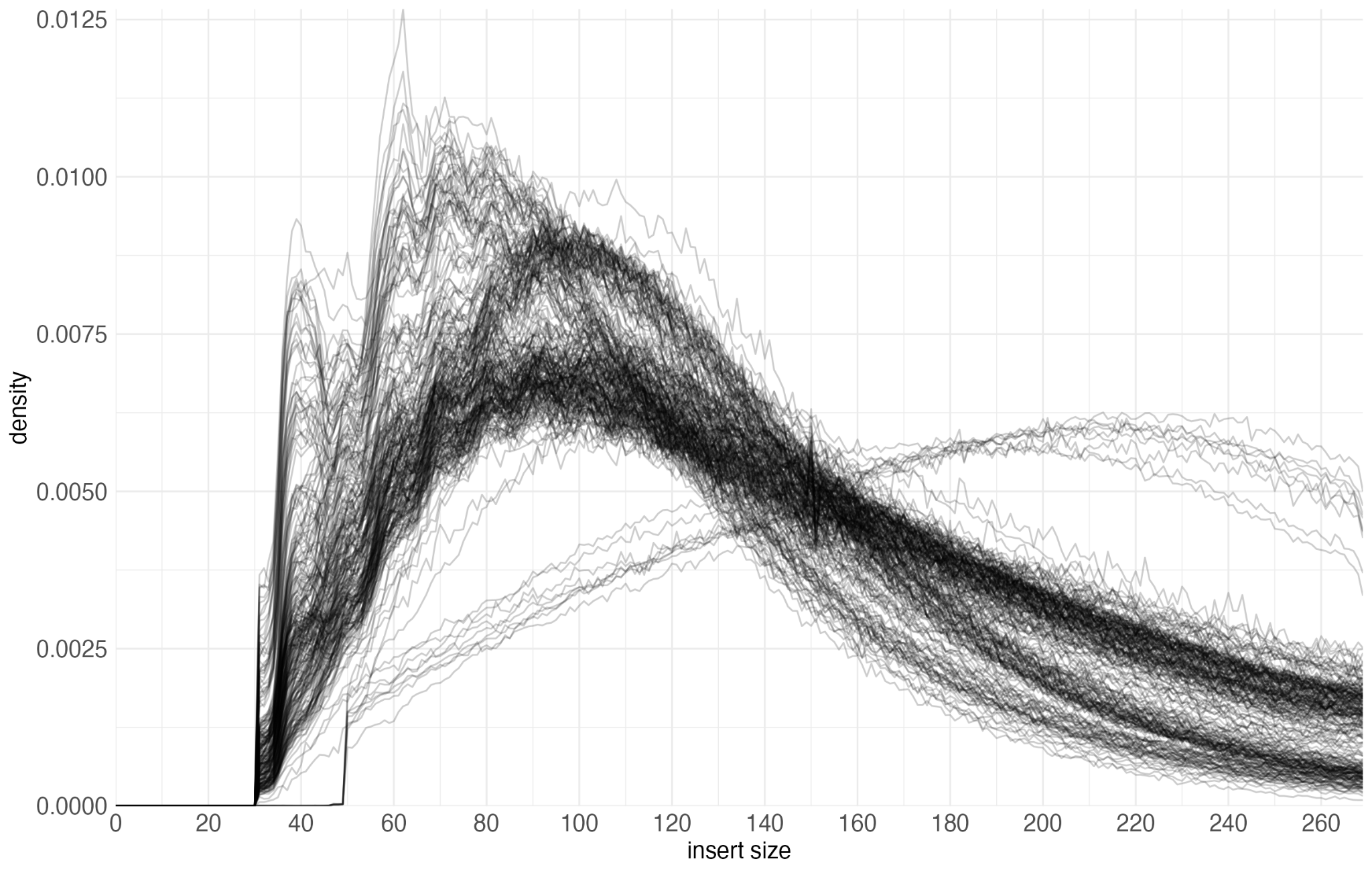


**Supplementary Figure 5.** Read length distributions for soil metagenomes from outlier metagenomes (**Supplementary Note 3**).

**
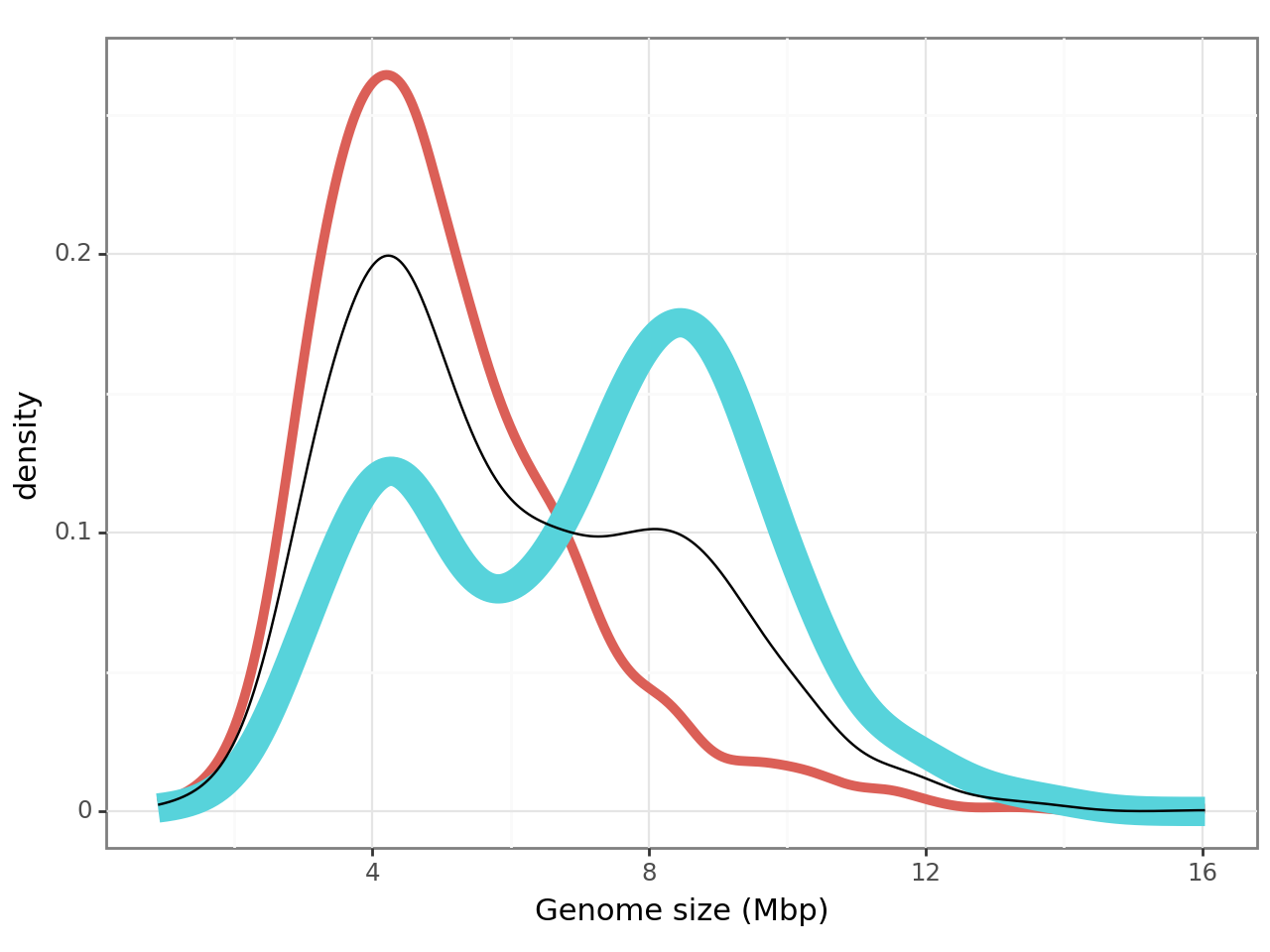
**

**Supplementary Figure 6.** Genome sizes of species isolated from soil. The thinnest black line represents all species and shows a bimodal distribution. The aqua line represents species from the phylum Actinomycetota, while the medium-thinness salmon line represents all other species combined. The y-axis represents the density of species as calculated using the “geom_density” function of ggplot2 [(Wickham 2016)](https://paperpile.com/c/64oTyf/Gm6F).

## References

[Hevroni, Gur, José Flores-Uribe, Oded Béjà, and Alon Philosof. 2020. “Seasonal and Diel Patterns of Abundance and Activity of Viruses in the Red Sea.” *Proceedings of the National Academy of Sciences of the United States of America* 117 (47): 29738–29747.](http://paperpile.com/b/64oTyf/jUmH)

[Hugerth, Luisa W., John Larsson, Johannes Alneberg, et al. 2015. “Metagenome-Assembled Genomes Uncover a Global Brackish Microbiome.” *Genome Biology* 16 (December): 279.](http://paperpile.com/b/64oTyf/9O7l)

[Ma, Bin, Caiyu Lu, Yiling Wang, et al. 2023. “A Genomic Catalogue of Soil Microbiomes Boosts Mining of Biodiversity and Genetic Resources.” *Nature Communications* 14 (1): 7318.](http://paperpile.com/b/64oTyf/BIfN)

[Pascoal, Francisco, Maria Paola Tomasino, Roberta Piredda, et al. 2023. “Inter-Comparison of Marine Microbiome Sampling Protocols.” *ISME Communications* 3 (1): 84.](http://paperpile.com/b/64oTyf/ESMf)

[Riley, Robert, Robert M. Bowers, Antonio Pedro Camargo, et al. 2023. “Terabase-Scale Coassembly of a Tropical Soil Microbiome.” *Microbiology Spectrum* 11 (4). https://doi.org/](http://paperpile.com/b/64oTyf/RfTu)[10.1128/spectrum.00200-23](http://dx.doi.org/10.1128/spectrum.00200-23)[.](http://paperpile.com/b/64oTyf/RfTu)

[Schober, Isabel, Julia Koblitz, Joaquim Sardà Carbasse, et al. 2025. “BacDive in 2025: The Core Database for Prokaryotic Strain Data.” *Nucleic Acids Research* 53 (D1): D748–D756.](http://paperpile.com/b/64oTyf/sm94)

[Sunagawa, Shinichi, Luis Pedro Coelho, Samuel Chaffron, et al. 2015. “Ocean Plankton. Structure and Function of the Global Ocean Microbiome.” *Science* 348 (6237): 1261359.](http://paperpile.com/b/64oTyf/UtNb)

[Wickham, Hadley. 2016. *ggplot2: Elegant Graphics for Data Analysis*. Springer International Publishing.](http://paperpile.com/b/64oTyf/Gm6F)

[Woodcroft, Ben J., Samuel T. N. Aroney, Rossen Zhao, et al. 2025. “Comprehensive Taxonomic Identification of Microbial Species in Metagenomic Data Using SingleM and Sandpiper.” *Nature Biotechnology*, July 16, 1–6.](http://paperpile.com/b/64oTyf/zSVS)
